# Editorial: The Role of Non-stoichiometry in the Functional Properties of Oxide Materials

**DOI:** 10.3389/fchem.2019.00547

**Published:** 2019-08-08

**Authors:** Claudio Cazorla, Maria Verónica Ganduglia-Pirovano, Javier Carrasco

**Affiliations:** ^1^School of Materials Science and Engineering, University of New South Wales, Sydney, NSW, Australia; ^2^Institute of Catalysis and Petrochemistry-CSIC, Madrid, Spain; ^3^CIC Energigune, Vitoria-Gasteiz, Spain

**Keywords:** oxide materials, density functional theory, experiments, oxygen vacancies, functionality

Oxides can show a plethora of emergent complex phenomena beyond their traditional role as dielectrics, which makes them attractive materials to a very broad range of scientific and technological processes. This includes areas such as catalysis, energy conversion and storage, and magnetism, to name just a few. Furthermore, nano-engineered oxides offer the possibility of realizing hitherto unobserved phenomena as due to their unique responsiveness to external stimuli, multiple order-parameter couplings, and increased volume-to-surface ratio. Yet, controlled synthesis and thorough microscopic understanding of functional oxide materials remain challenging. This is a complex task to accomplish due to the ubiquitous stoichiometry fluctuations found in oxides, which affect considerably their structural, chemical, and magnetic properties (Venkatesan et al., 2004; Herklotz et al., 2017).

Non-stoichiometry is indeed a pivotal facet of the functioning of oxide materials. For instance, the presence of oxygen vacancies in oxide-based heterogeneous catalysts is often necessary for any activity (Campbell and Peden, 2005; Jeen et al., 2013). Oxygen vacancies can also distort significantly the equilibrium arrangement of atoms and modify the super-exchange interactions between neighboring magnetic ions, thus inducing spin-order transformations (Wang et al., 2003; Eerenstein et al., 2005). Furthermore, extrinsic vacancies enable ionic conductivity in perovskite-based solid solutions to be used in electrochemical applications such as solid oxide fuel and electrolytic cells (Adler, 2004; Chroneos et al., 2011). Engineering of chemical defects in oxide compounds, therefore, emerges as a likely avenue for the design of new materials with tailored functionality. In this Research Topic, we present a collection of original articles that highlight how state-of-the-art theoretical and experimental approaches are leading to a better atomic-level understanding of non-stoichiometric oxide systems and improved design of novel functional materials.

From a theoretical viewpoint, the accurate description of the electronic structure of non-stoichiometric oxides of technological interest is not an easy task and requires efficient and robust theory (Ganduglia-Pirovano et al., 2007). Martínez-Casado et al. thoroughly analyze this issue for defective anatase surfaces and conclude that all-electron-based hybrid density functionals are required to yield accurate trends in the position of band gap states of defects and adsorbates in large model systems. Furthermore, Poli et al. efficiently combine non-local density functional theory (DFT) and linear-response time-dependent DFT to enable the interpretation of recent experimental UV-vis spectra for cation-vacancy defects in aluminosilicate imogolite nanotubes, considering large supercells of around 1,000 atoms.

Research on non-stoichiometric oxide materials is typically focused on bulk and extended surfaces. Generally, the interaction of oxygen vacancies in such materials is poorly understood. Gao et al. address the mechanisms of aggregation of oxygen vacancies in SiO_2_ and HfO_2_ theoretically and suggest that electron trapping at oxygen vacancies may facilitate the creation of new neighboring vacancies and that this process becomes even more efficient with increasing vacancy cluster size. This finding is particularly relevant for improving the performance of such materials in applications where, for example, electrons can be injected via tunneling from an electrode in a device. At non-stoichiometric oxide surfaces, the structure and dynamics of the near-surface oxygen vacancies and excess charges are most relevant for technological applications such as catalysis. Wolf et al. address the heavily discussed appearance and properties of anionic defects at the (111)-oriented CeO_2_ ceria surface in scanning tunneling microscopy (STM) images, and Han et al. investigate the effect of applied stress on the relative stability of isolated surface and subsurface oxygen vacancies at the same surface, the formation of vacancy pairs, and the localization of the excess charge. Noticeably, at moderate compressive strains the formation of first-neighbor surface oxygen vacancy pairs is energetically preferred over that of isolated vacancies, contrary to what it is predicted at null strain. Yet, little is known about nanosize effects on oxygen vacancy formation. This salient aspect of current materials design is theoretically explored by Cuko et al. considering as a case study titania, silica, and mixed Ti_x_Si_1−x_O_2_ nanoclusters. Interestingly, the authors show that, in contrast to the results obtained for the bulk and for surfaces, silica nanoclusters are more reducible than titania, thus disclosing an unexpected chemical behavior at the nanoscale. Moreover, Rhatigan and Nolan model hydroxylated titania surfaces modified with dispersed Mn_4_O_x_ nanoclusters and reveal important changes in light absorption and reactivity toward water splitting as a consequence of the formation of nanocluster-surface interactions that influence the system electronic structure. Furthermore, Kim et al. consider the effect that agglomeration of CeO_2_ nanoparticles have on the stability and oxygen vacancy formation energy, applying a computational protocol based on a multiscale simulation approach. Surprisingly, for a possible {100}/{100} interface of two agglomerated Ce_40_O_80_ truncated octahedral, the energy cost to create an oxygen vacancy is negative, indicating that under reducing conditions, oxygen removal from the interface will have a stabilizing effect.

The structural changes of oxide surfaces during redox processes are essential to understanding their properties in many applications. Schöttner et al. track the evolution of the hematite a-Fe_2_O_3_(0001) surface under reducing conditions by polarization-resolved infrared reflection absorption spectroscopy (IRRAS) using CO as a probe molecule. Not only the formation of oxygen vacancies is observed, but a massive reconstruction yielding the formation of Fe_3_O_4_(111) domains. Such findings cannot be ignored when thinking about the surface chemistry of iron oxide surfaces. Furthermore, Luches et al. focus on the dynamics of the interaction between ceria epitaxial islands and films on Pt (111) during reduction and oxidation, and observe different reduced structural phases that include the formation of a Ce-Pt alloy. These results are most relevant for the understanding of Pt/CeO_2_ catalysts, which can be used for low-temperature CO oxidation, as discussed by Kardash et al., who investigate Pt/CeO_x_ and Pt/CeSnO_x_ systems by means of a synergistic approach that combines experiment and theory. It is found that the Sn-doping promotes oxygen removal and increases catalytic activity. The effect of catalyst doping is also considered by Alvarez-Galvan et al., who address the production of synthesis gas from methane over Rh-doped Ni-based catalysts, supported on Al_2_O_3_, CeO_2_, La_2_O_3_, MgO, and ZrO_2_. This is an important process for converting natural gas into added value high-quality liquid products. However, current catalysis methods for achieving partial oxidation of methane are energy-intensive and hence more energy-efficient alternatives to produce synthesis gas are highly desirable. It is found that Ni/CeO_2_ catalyst shows the best behavior per surface area and that small amount of Rh improves the catalytic performance.

We believe that the Role of Non-stoichiometry in the Functional Properties of Oxide Materials Research Topic provides recent insights into our ongoing quest for rationalizing the intriguing phenomena observed in non-stoichiometric oxide systems and how this can affect their functionality, with special focus on energy harvesting applications.

## Author Contributions

All authors listed have made a substantial, direct and intellectual contribution to the work, and approved it for publication.

### Conflict of Interest Statement

The authors declare that the research was conducted in the absence of any commercial or financial relationships that could be construed as a potential conflict of interest.
